# Nuggets

**Published:** 1891-04

**Authors:** C. Carleton Smith


### NUGGETS.

### An Answer to Dr. Lilienthal.

With regard to the conditions of aggravation and amelioration of Phos., better lying on left side and worse lying on right side, I would reply that I obtained them many years ago from my departed friend and counsellor, Constantine Hering. And would add that, later on, these were verified by the late Dr. R. R. Gregg, of Buffalo. Aggravation from least pressure on intercostal muscles, I made a marginal note of some twenty years ago after curing a little girl of an affection of the right lung with Phos. where that symptom was prominent, after a leading allopathic physician had given the case up and requested me to step in and take charge. However, aggravation in a given case from lying on right side would not necessarily exclude Phos. if the other symptoms agreed with that drug; for, as you wisely remark, "I think Smith and Hering right when we take the pathological condition in view which causes this aggravation or amelioration, because the patient needs all the air and oxygen he can get to breathe, and will prefer *that position*

favorable to easier breathing," which observation is undoubtedly the true state of the case.

The holding of the abdomen with the hands when the coughing spells come on is not, according to my observation, for the purpose of relief from *pressure*, but merely to prevent further pain from the bulging out of the abdominal walls occasioned by the concussion accompanying each paroxysm of cough, for the very patient who, in a quiescent state will not allow pressure on the walls of the abdomen, will instantly place his hands over the same locality when the cough comes on. But the action is merely for support, nothing else.

Very faithfully yours,

C. Carleton Smith.

